# Urinary uromodulin independently predicts end-stage renal disease and rapid kidney function decline in a cohort of chronic kidney disease patients

**DOI:** 10.1097/MD.0000000000015808

**Published:** 2019-05-24

**Authors:** Dominik Steubl, Matthias Block, Victor Herbst, Wolfgang Andreas Nockher, Wolfgang Schlumberger, Stephan Kemmner, Quirin Bachmann, Susanne Angermann, Ming Wen, Uwe Heemann, Lutz Renders, Pranav S. Garimella, Jürgen Scherberich

**Affiliations:** a Abteilung für Nephrologie, Klinikum rechts der Isar, Fakultät für Medizin, Technische Universität München, München; b Euroimmun Medizinische Labordiagnostika AG, Lübeck; c Institut für Laboratoriumsmedizin und Pathobiochemie, Molekulare Diagnostik, Universitätsklinikum Marburg, Philipps-Universität Marburg, Marburg, Germany; d Division of Nephrology and Hypertension, University of California San Diego, San Diego, CA; e Klinikum München-Harlaching, München, Germany.

**Keywords:** biomarker, CKD, decline, eGFR, ESRD, predictor, Tamm–Horsfall protein, uromodulin

## Abstract

Data on risk factors predicting rapid progression to end-stage renal disease (ESRD) or short-term kidney function decline (i.e., within 1 year) in chronic kidney disease (CKD) are rare but urgently needed to plan treatment. This study describes the association and predictive value of urinary uromodulin (uUMOD) for rapid progression of CKD.

We assessed uUMOD, demographic/treatment parameters, estimated glomerular filtration rate (eGFR), and proteinuria in 230 CKD patients stage I-V. ESRD and 25% decline of eGFR was documented at the end of follow-up period and used as a composite endpoint. Association between logarithmic uUMOD and eGFR/proteinuria was calculated using linear regression analysis, adjusting for age, gender, and body mass index. We performed multivariable Cox proportional hazard regression analysis to evaluate the association of uUMOD with the composite endpoint. Therefore, patients were categorized into quartiles. The predictive value of uUMOD for the above outcomes was assessed using receiver-operating characteristic (ROC) curve analysis.

Follow-up was 57.3 ± 18.7 weeks, baseline age was 60 (18;92) years, and eGFR was 38 (6;156) mL/min/1.73 m^2^. Forty-seven (20.4%) patients reached the composite endpoint. uUMOD concentrations were directly associated with eGFR and inversely associated with proteinuria (β = 0.554 and β = -0.429, *P* < .001). In multivariable Cox regression analysis, the first 2 quartiles of uUMOD concentrations had a hazard ratio (HR) of 3.589 [95% confidence interval (95% CI) 1.002–12.992] and 5.409 (95% CI 1.444–20.269), respectively, in comparison to patients of the highest quartile (≥11.45 μg/mL) for the composite endpoint. In ROC-analysis, uUMOD predicted the composite endpoint with good sensitivity (74.6%) and specificity (76.6%) at an optimal cut-off at 3.5 μg/mL and area under the curve of 0.786 (95% CI 0.712–0.860, *P* < .001).

uUMOD was independently associated with ESRD/rapid loss of eGFR. It might serve as a robust predictor of rapid kidney function decline and help to better schedule arrangements for future treatment.

## Abstract 2

Supplemental Digital Content is available in the text

## 1 Introduction

Chronic kidney disease (CKD) represents one of the major medical burdens in Western countries. Health care costs associated with CKD are high and further increase when end-stage renal disease (ESRD) is reached.^[1–3]^ In addition, morbidity and mortality are significantly elevated, predominantly due to cardiovascular complications.^[4]^ Therefore, it is crucial to diagnose patients with CKD early and to identify those who have rapid CKD progression to potentially intervene or prepare them for renal replacement therapy.^[5–7]^ Biomarkers appear to be an attractive diagnostic approach to early identify CKD patients and those who are at risk for rapid CKD progression.^[8,9]^ Different parameters for the prediction of ESRD or decline of estimated glomerular filtration rate (eGFR) and death in the long term (>3 years of follow-up) have been evaluated.^[10–16]^ However, markers that predict kidney function decline in the short term are needed in order to take measures such as hemodialysis access. Recently, urinary uromodulin (uUMOD) has been identified as a valuable parameter for the prediction of ESRD and progression of CKD in a large cohort over a period >9 and >3 years, respectively.^[17,18]^

In this study, we evaluated whether uUMOD is associated with rapid loss of eGFR and ESRD in CKD patients within 1 year of follow-up.

## 2 Patients and methods

The final cohort consisted of 230 patients at stages I-V of CKD who presented to the outpatient clinic of a tertiary care university hospital. The study was approved by the local ethics committee of Klinikum rechts der Isar, Technische Universität, Munich, Germany, and adheres to the declaration of Helsinki. All patients enrolled in this study gave their informed consent. The only inclusion criteria followed the definitions for CKD according to the last KDIGO guidelines^[19]^: “CKD is defined as abnormalities of kidney structure or function, present for **>**3 months, with implications for health.” Therefore, we established the diagnosis of CKD when either eGFR was <60 mL/min and/or apparent signs of kidney damage were present over a period of 3 months. As apparent signs of kidney damage, we considered proteinuria with a cut-off >150 mg/g creatinine on spot urine specimen and/or histologically proven kidney disease and/or abnormalities detected in imaging techniques (ultrasound, computed tomography, magnetic resonance imaging, or nuclear imaging). Calculation of eGFR was based on both serum creatinine and cystatin C concentrations (CKD-EPI_crea-cystatin_).^[20]^ Exclusion criteria were age <18 years, psychiatric comorbidities that would not allow written informed consent, and lack of serum/urine sample at the time of potential enrollment. Furthermore, patients with symptomatic or asymptomatic urinary tract infection (UTI), defined as detection of leucocytes and/or bacteria in the urinary sediment at the time of enrollment, were excluded, as it is unclear how acute UTI affects uUMOD secretion. The following parameters were assessed, as they were shown to be relevant markers for CKD progression^[21–24]^: eGFR, systolic blood pressure (SBP), diastolic blood pressure (DBP), spot proteinuria (calculated as mg/g urine creatinine), and C-reactive protein (CrP). We compiled the following demographic variables: age, gender, body mass index (BMI), accompanying coronary heart disease (CHD), peripheral artery disease (PAD), and concomitant diabetes mellitus (data were obtained from electronic chart review). Prevalence of CHD and PAD was recorded according to the last medical report, concomitant diabetes in view of the antidiabetic medication and/or HbA1c levels above the cut-off of 5.9%. All laboratory measurements, except uUMOD, had been performed at the day of enrollment. Medications with a renoprotective effect were recorded, including angiotensin-converting enzyme (ACE)-inhibitors/AT1-antagonists, aldosterone antagonists, bicarbonate, erythropoietin, uric acid lowering agents, active vitamin D, and phosphate binding agents.

Primary outcome was reaching ESRD or a 25% decline in eGFR as a composite endpoint within 1 year of follow-up (ascertained through chart review). We chose the 25% cut-off relying on recent studies that demonstrated the ability of a lower eGFR decline to predict renal outcome.^[25,26]^ Follow-up was obtained 12 months after inclusion of the last patient into the study.

Patients’ demographics, medication, and predictive parameters are presented in Table 1.

**Table 1:**
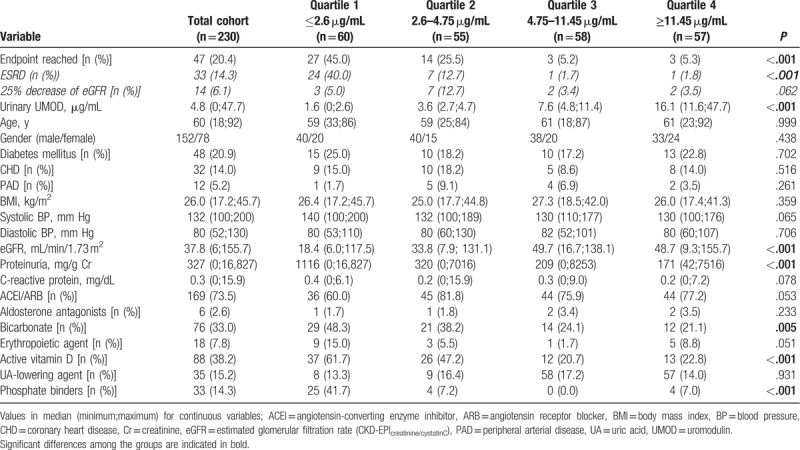
Baseline characteristics of the study population and quartiles according to urinary uromodulin concentrations.

### 2.1 Measurement of urinary uromodulin

All urine samples were stored at –80°C before measurements were performed. Urinary uromodulin measurements were performed using a commercially available assay (Euroimmun AG, Lübeck, Germany). Short performance characteristics of the ELISA for plasma samples given by the manufacturer are as follows: detection limit for plasma samples 2 ng/mL; mean linearity recovery 97% (83–107% at 59–397 ng/mL); intra-assay precision 1.8–3.2% (at 30–214 ng/mL), inter-assay precision 6.6–7.8% (at 35–228 ng/mL), and inter-lot precision 7.2–10.1% (at 37–227 ng/mL). Urine samples were diluted 1 : 101 using dilution buffer. One hundred microliter of calibrators, controls, or diluted samples were pipetted into coated wells of the microtiter plate (MTP); subsequently, 100 μL of biotinylated detection antibody (final concentration 50 ng/mL) were added. The MTP was covered with foil and incubated for 2 hours at 450 rotations per minute (rpm) and room temperature on a rotary shaker. After 2 hours, the MTP was washed 3 times using 300 μL washing buffer, and then the wells were tapped gently. One hundred microliter of Steptavidin-Polyperoxidase (SPO, final concentration 67 ng/mL) were pipetted into each well followed by another incubation for 30 minutes at 450 rpm. Subsequently, the SPO was soaked and the MTP washed 3 times with 300 μL of washing buffer. Consequently, 100 μL of substrate solution (containing the chromogen tetramethylbenzidin and hydrogen peroxide as the substrate for SPO) were pipetted into each well. The MTP was incubated in the dark for 15 minutes at room temperature. The reaction was terminated by adding 100 μL of stop solution. This causes a color change from blue to yellow. Finally, the substrate solution was measured using a photometer at a wavelength of 450 nm and reference wavelength of 620 nm. Data analysis was performed using the program Magellan (Tecan Group Ltd., Männedorf, Switzerland).

### 2.2 Statistics

Due to skewed distribution, data are presented as median with minimum and maximum. Categorical variables are reported in absolute numbers and percentages. We evaluated the correlations of uUMOD, eGFR, and proteinuria using linear regression modeling adjusted for age, gender, and BMI. To better fit the model, we log transformed uUMOD, eGFR serum concentrations, and proteinuria. Subsequently, we divided the cohort into quartiles according to uUMOD concentrations for further analysis. For comparison of demographic data, medication, and laboratory parameters between the quartiles, exact Fisher test for categorical variables and Kruskal–Wallis test for continuous variables were used. Univariable Cox regression analysis was calculated for each variable with the composite endpoint being the dependent variable and the predictor being the independent one. For further work-up, we chose a stepwise approach: parameters significantly associated with the endpoint in univariable analysis (*P* < .05) were included in the multivariable analysis using forward inclusion. uUMOD was further evaluated in receiver-operating curve (ROC)-analysis to assess the cut-off point (OCO) with optimal sensitivity and specificity to predict the composite endpoint. Kaplan–Meier analysis was performed to illustrate the association between uUMOD and the composite endpoint.

All reported *P* values are 2-sided, with a significance level of .05 and have not been adjusted for multiple testing. For statistical analysis, SPSS 23 (IBM, Armonk, NY) was used.

## 3 Results

### 3.1 Patients’ demographics

Three hundred five patients were initially included in the study. At the time of follow-up assessment, 75 (24.6%) patients were lost to follow-up. The patients did not differ substantially from the remaining 230 patients included in the final analysis (Suppl. Table 1 vs Table 1). The mean age of the subjects included was 60 (minimum 18; maximum 92) years, and 152 (66%) were male. Glomerulonephritis was the most frequent underlying disease (UD) with 87 of 230 patients (37.8%, Table 2). Forty-eight (20.9%) patients suffered from diabetes mellitus, which was the cause for CKD in 17 (7.4%) patients (Table 2). In 31 (13.5%) patients, arterial hypertension was the underlying cause for CKD (Table 2). The number of patients within each CKD stage were as follows: 22 (9.6%) stage I, 39 (14.4%) stage II, 82 (35.7%) stage III, 56 (20.7%) stage IV, 31 (11.5%) stage V.

**Table 2:**
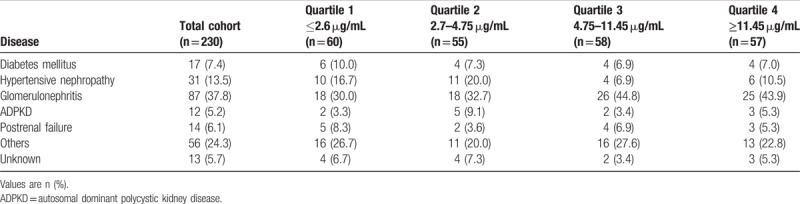
Causes for end-stage renal disease in the total cohort and urinary uromodulin quartiles.

Detailed baseline characteristics of the participants are presented in Table 1. The classification of UD is reported in Table 2.

Forty-seven (20.4%) patients reached the composite endpoint, of whom 33 patients reached ESRD and 14 experienced at least 25% decrease in eGFR but not ESRD (Table 1). Of the patients reaching ESRD, 2 were stage CKD III, 12 CKD IV, and 19 CKD V. Among the patients only experiencing at least 25% decrease in eGFR but not ESRD, the patients were widely distributed among all stages of CKD: 2 patients stage I, 3 patients stage II, 3 patients stage III, 5 patients stage IV, and 1 patient stage V.

The composite endpoint was reached by 27 (57.4% of all patients reaching the endpoint) patients of quartile 1 (uUMOD ≤2.6 μg/mL), 14 (29.8%) of quartile 2 (uUMOD 2.6–4.75 μg/mL), 3 (6.4%) of quartile 3 (4.75–11.45 μg/mL), and 3 (6.4%) of quartile 4 (uUMOD ≥11.45 μg/mL, Table 1).

**Figure 1:**
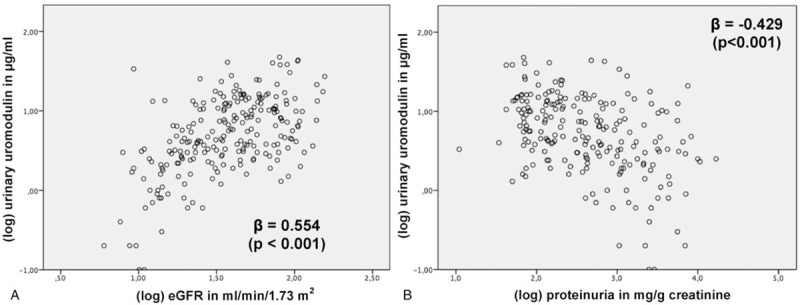
Multivariable linear regression analysis to evaluate the association between logarithmic (log) urinary uromodulin and (A) (log) estimated glomerular filtration rate (eGFR), (B) (log) proteinuria; analysis adjusted for age, gender, and body mass index.

In multivariable linear regression analysis, (log) uUMOD and (log) eGFR showed a significant positive association (β = 0.554, *P* < .001, Fig. 1). The association between (log) uUMOD and (log) proteinuria was at a similar, but inverse level (β = -0.429, *P* < .001, Fig. 1).

### 3.2 Univariable analysis of differences between uUMOD quartiles

Demographic parameters did not differ significantly between the quartiles (Table 1). The quartile with the lowest uUMOD concentrations had the lowest eGFR and the highest degree of proteinuria (*P* < .001), the latter decreasing to the quartile with the highest uUMOD concentrations (Table 1). CRP was not different between the groups. The quartile with the lowest uUMOD concentrations had a significantly higher proportion of bicarbonate (*P* = .005), active vitamin D, and phosphate binding medication (*P* < .001, Table 1). ACE-inhibitors/ARBs, erythropoiesis-stimulating, and uric acid lowering agent prescription were not statistically different within the quartiles (Table 1).

### 3.3 Univariable and multivariable Cox proportional hazard regression analysis

**Figure 2:**
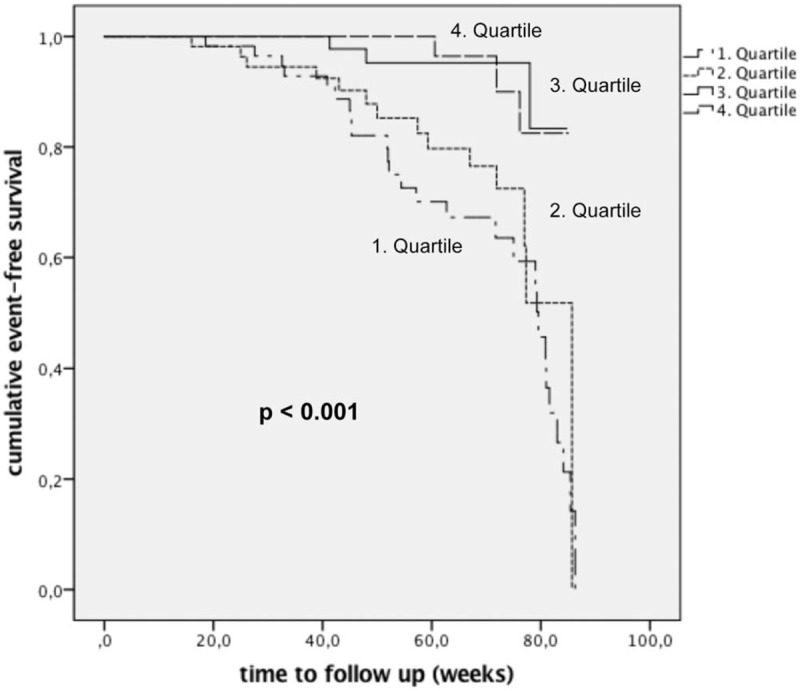
Kaplan–Meier curves of the composite endpoint (end-stage renal disease and/or >25% decrease of estimated glomerular filtration rate during follow-up) of 230 chronic kidney disease patients, classified into 4 quartiles according to their urinary uromodulin concentrations at baseline: quartile ≤2.6 μg/mL, quartile 2.6–4.75 μg/mL, quartile 4.75–11.45 μg/mL, and quartile ≥11.45 μg/mL. The quartiles with lower uromodulin concentrations exhibited a significantly higher risk to reach the endpoint than the other 2 quartiles (log-rank test, *P* < .001).

In univariable Cox regression analysis, uUMOD concentrations of the 2 lower quartiles (≤2.6 and 2.7–4.75 μg/mL) were associated with an HR of 6.362 (95% CI 1.906–21.234) and 4.600 (95% CI 1.320–16.031) to reach the composite endpoint in comparison to the reference quartile with the patients having the highest uUMOD concentrations (Table 3). Furthermore, systolic BP (HR 1.017 per mmHg higher, 95% CI 1.002–1.032), eGFR (HR 0.976 per mL/min/1.73 m^2^ higher, 95% CI 0.960–0.992), proteinuria (HR 1.018 per 100 mg/g creatinine higher, 95% CI 1.011–1.025), CrP (HR 1.172 per mg/dL higher, 95% CI 1.040–1.320), oral active vitamin D (HR 2.523, 95% CI 1.279–4.977), and phosphate-binding agents use (HR 4.092, 95% CI 2.253–7.432) were associated with the endpoint in univariable analysis (Table 3). After adjusting for these variables in multivariable Cox regression analysis, the 2 lowest quartiles were still independently associated with the composite endpoint: the group with the lowest uUMOD concentrations showed a HR of 3.589 (95% CI 1.002–12.992), and the second lowest quartile even had a higher HR (HR 5.409, 95% CI 1.444–20.269). Likewise, in Kaplan–Meier curve analysis, the patients of the 2 lower quartiles had a significantly higher risk to reach the composite endpoint (log-rank test *P* < .001, Fig. 2). Finally, in multivariable analysis, we did not detect any significant interactions between uUMOD quartiles and eGFR/proteinuria (suppl. Table 2).

**Table 3:**
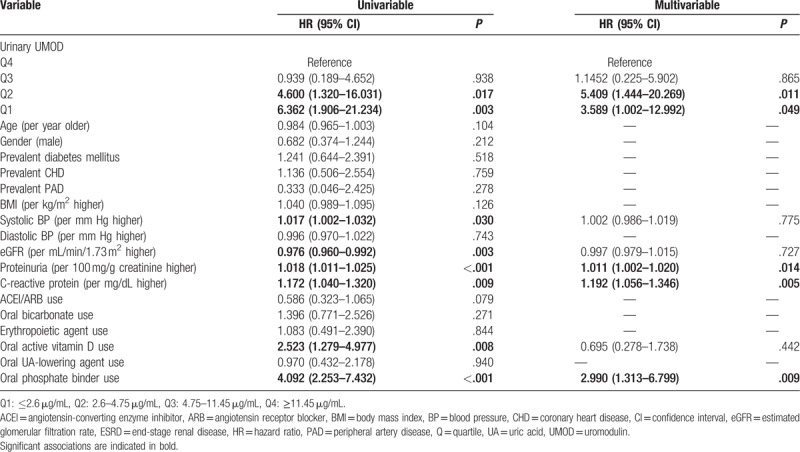
Univariable and multivariable cox proportional hazard regression analysis with the composite endpoint ESRD/25% eGFR decline.

### 3.4 ROC-analysis

**Figure 3:**
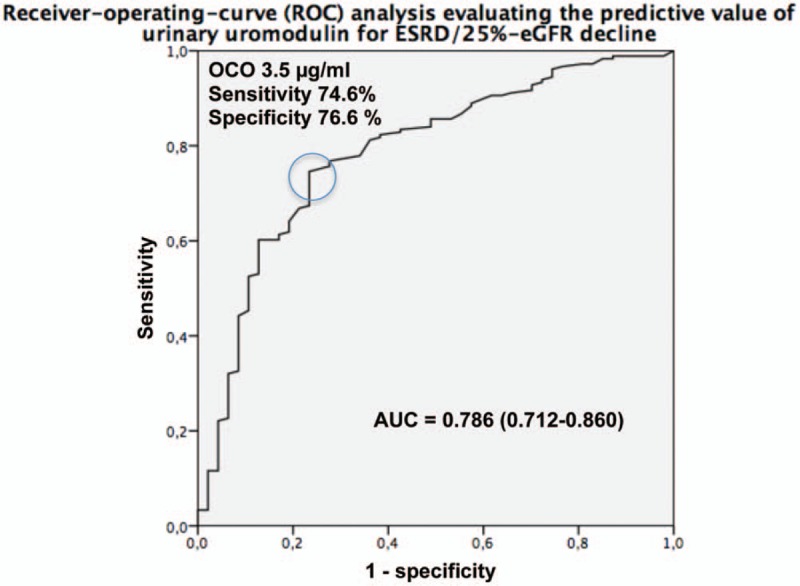
ROC-analysis evaluating the predictive value of urinary uromodulin for ESRD/25% eGFR decline. AUC = area under the curve, eGFR = estimated glomerular filtration rate, OCO = optimal cut-off.

In ROC-analysis, uUMOD [area under the curve (AUC) 0.786, 95% CI 0.712–0.860, *P* < .001, Fig. 3] discriminated the endpoint with a sensitivity of 74.6% and specificity 76.6% at an OCO of 3.5 μg/mL.

## 4 Discussion

In order to take necessary steps to treat CKD patients (e.g., to prepare the patient for renal replacement therapy), biomarkers that predict rapid deterioration of kidney function are needed, but data on this topic are very rare. uUMOD has been shown to predict development of CKD over a period of >9 years.^[18]^ To our knowledge, we demonstrated here for the first time that uUMOD is also associated with rapid progression to ESRD and/or rapid decline of kidney function within 1 year.

uUMOD is discussed to play a pathogenic role in CKD.^[27]^ uUMOD has been only moderately well correlated to eGFR previously.^[18,28]^ We detected a moderate association between logarithmic uUMOD and eGFR in our cohort. This suggests that uUMOD potentially represents tubular mass independently from glomerular function, as urinary uromodulin excretion has been shown to correlate with tubular mass.^[29]^ Furthermore, luminal secretion of UMOD into the urine appears to be differently regulated from apical release, as circulatory UMOD was shown to nominally correlate stronger to eGFR than uUMOD.^[30]^ Besides this, tubular mass appears to be important for the preservation of overall kidney function, as we detected that loss of kidney function was predicted by uUMOD independently from eGFR. The question if the predictive value of uUMOD is based on pathophysiologic mechanisms or simply by reflection of tubular mass is beyond the scope of this article.

Other urinary markers were evaluated with regard to their predictive value for loss of kidney function. Urinary neutrophil gelatinase associated lipocalin (NGAL) concentrations in combination with urinary creatinine concentrations were associated with rapid loss of renal function and ESRD in a cohort of 158 patients at stage 3 and 4 of CKD.^[31]^ However, a larger study on >3000 patients did not show a substantial benefit of urinary NGAL concentrations as a predictor when added to known parameters such as proteinuria in CKD patients for adjustment.^[16]^ Urinary cystatin C has not been extensively studied in this context. A Korean study proved its value only in normoalbuminuric diabetic patients.^[32]^ Urinary kidney injury molecule 1 (KIM-1) was also evaluated for predicting CKD progression.^[33–35]^ Bhavsar et al^[33]^ and Nielsen et al^[35]^ could not demonstrate a benefit using KIM-1 for risk stratification. Similarly, the value of KIM-1 as a predictor was also rather limited in the study of Peralta et al,^[34]^ only showing a significant difference when comparing the highest decile with the lower 90% of patients. Another problem is that urinary KIM-1 is significantly influenced by medication and sodium restriction.^[36]^ As KIM-1 is suggested to be a marker for acute tubular injury (e.g., prolonged ischemia), KIM-1 appears to be rather useful in the setting of acute kidney failure.^[37,38]^

Recent research focused on a urinary proteomic analysis approach to predict the risk of CKD progression.^[39]^ Although this appears to be a promising way to identify alterations of renal cell activity, interaction, and loss of renal tissue in a dynamic analysis, we currently consider this method a long way off clinical practice due to very high costs. In addition, further reliable data are needed.

Wilson et al^[40]^ suggested a simple approach to estimate the risk for ESRD in CKD patients from urinary creatinine adjusted for fat-free mass. Although a significant predictive value of urinary creatinine was seen, the study did not adjust for parameters that we would assume to be of relevance such as concomitant pharmacologic treatment. Di Micco et al^[41]^ also suggested that lower urinary creatinine concentration predicted ESRD in stage 3 to 5 of CKD. However, a very moderate relationship was seen in multivariable analysis with an increased risk of 2% with every 20 mg/dL reduction of urinary creatinine concentration. Furthermore, no significant differences of urinary creatinine concentrations were seen in patients at CKD stage 5, hampering the use of urinary creatinine as a tool for risk evaluation within this important subcohort. eGFR and albuminuria were evaluated in a large meta-analysis with over 20,000 patients.^[42]^ Both parameters were predictive for ESRD, but the heterogeneity concerning eGFR was quite large between the studies. As our data suggest, eGFR might only be of value for risk stratification over a longer period. Proteinuria/albuminuria is indeed a helpful parameter that can also be influenced by clinical measures, but in the study of Astor et al,^[42]^ an 8-fold elevation of proteinuria was needed to reach a HR of 3.04. As the range in uUMOD was smaller in our study, uUMOD might be more promising, as also more subtle differences might indicate a change in risk.

Our study has limitations: we only analyzed the short-term outcomes, so no data on long-term relevance of uUMOD can be provided. However, this was already demonstrated by Garimella et al.^[18]^ Furthermore, data cannot be generalized, as we predominantly involved Caucasian patients. Also, a disproportional large percentage of patients included had glomerulonephritis as the UD, which does not fully represent the overall CKD population. Furthermore, over 30% of the patients included were at CKD stage IV-V, limiting the generalizability of the results to earlier CKD stages. Data were assessed in a single center, so local particularities could have an impact on the results. Samples were stored at -80°C before measurements were performed.

In conclusion, uUMOD appears to be a promising independent biomarker for risk stratification of rapid disease progression in CKD patients.

## Acknowledgments

We thank Dr Anna-Lena Hasenau, MD, for excellent logistic support.

## Author contributions

(1) Conception or design, or analysis and interpretation of data, or both: DS, MB, VH, PSG, JS.
(2) Collection of data and/or samples: DS, SK, QB, SA, MW.
(3) Drafting the article or revising it: DS, MB, VH, PSG, JS.
(4) Providing intellectual content of critical importance to the work described: WAN, WS, UH, LR, PSG.

**Conceptualization:** Dominik Steubl, Andreas Wolfgang Nockher, Wolfgang Schlumberger, Jürgen Scherberich.

**Data curation:** Dominik Steubl, Stephan Kemmner, Quirin Bachmann, Ming Wen.

**Formal analysis:** Dominik Steubl, Matthias Block.

**Investigation:** Dominik Steubl.

**Methodology:** Matthias Block, Wolfgang Schlumberger.

**Project administration:** Victor Herbst.

**Software:** Dominik Steubl.

**Supervision:** Uwe Heemann, Lutz Renders, Pranav S. Garimella, Jürgen Scherberich.

**Validation:** Dominik Steubl.

**Writing – original draft:** Dominik Steubl, Susanne Angermann, Jürgen Scherberich.

**Writing – review & editing:** Dominik Steubl, Susanne Angermann, Pranav S. Garimella, Jürgen Scherberich.

## Supplementary Material

Supplemental Digital Content

## References

[1] Eriksson JK Neovius M Jacobson SH Healthcare costs in chronic kidney disease and renal replacement therapy: a population-based cohort study in Sweden. BMJ Open 2016;6:e012062. 10.1136/bmjopen-2016-012062 PMC5073563 27855091

[2] Wyld ML Lee CM Zhuo X Cost to government and society of chronic kidney disease stage 1-5: a national cohort study. Intern Med J 2015;45:741–7. 25944415 10.1111/imj.12797

[3] Kent S Schlackow I Lozano-Kuhne J What is the impact of chronic kidney disease stage and cardiovascular disease on the annual cost of hospital care in moderate-to-severe kidney disease? BMC Nephrol 2015;16:65. 25924679 10.1186/s12882-015-0054-0 PMC4424521

[4] Neovius M Jacobson SH Eriksson JK Mortality in chronic kidney disease and renal replacement therapy: a population-based cohort study. BMJ Open 2014;4:e004251. 10.1136/bmjopen-2013-004251 PMC3931988 24549162

[5] Black C Sharma P Scotland G Early referral strategies for management of people with markers of renal disease: a systematic review of the evidence of clinical effectiveness, cost-effectiveness and economic analysis. Health Technol Assess 2010;14:1–84. 10.3310/hta14210 20441712

[6] Perico N Remuzzi G Need for chronic kidney disease prevention programs in disadvantaged populations. Clin Nephrol 2015;83 7 suppl 1:42–8. 25725241 10.5414/cnp83s042

[7] National Clinical Guideline C. National Institute for Health and Care Excellence: Clinical Guidelines. Chronic Kidney Disease (Partial Update): Early Identification and Management of Chronic Kidney Disease in Adults in Primary and Secondary Care. London: National Institute for Health and Care Excellence.(UK). S Copyright (c) National Clinical Guideline Centre 2014; 2014.

[8] Nickolas TL Barasch J Devarajan P Biomarkers in acute and chronic kidney disease. Curr Opin Nephrol Hypertens 2008;17:127–32. 18277143 10.1097/MNH.0b013e3282f4e525

[9] Rysz J Gluba-Brzozka A Franczyk B Novel biomarkers in the diagnosis of chronic kidney disease and the prediction of its outcome. Int J Mol Sci 2017;18:pii: E1702. 10.3390/ijms18081702 PMC5578092 28777303

[10] Sim JJ Bhandari SK Smith N Phosphorus and risk of renal failure in subjects with normal renal function. Am J Med 2013;126:311–8. 23375678 10.1016/j.amjmed.2012.08.018

[11] Kestenbaum B Sampson JN Rudser KD Serum phosphate levels and mortality risk among people with chronic kidney disease. J Am Soc Nephrol 2005;16:520–8. 15615819 10.1681/ASN.2004070602

[12] Rahman M Yang W Akkina S Relation of serum lipids and lipoproteins with progression of CKD: the CRIC study. Clin J Am Soc Nephrol 2014;9:1190–8. 24832097 10.2215/CJN.09320913 PMC4078958

[13] Portoles J Gorriz JL Rubio E The development of anemia is associated to poor prognosis in NKF/KDOQI stage 3 chronic kidney disease. BMC Nephrol 2013;14:2. 23295149 10.1186/1471-2369-14-2 PMC3623844

[14] Nacak H van Diepen M de Goeij MC Uric acid: association with rate of renal function decline and time until start of dialysis in incident pre-dialysis patients. BMC Nephrol 2014;15:91. 24939671 10.1186/1471-2369-15-91 PMC4075499

[15] Agarwal R Duffin KL Laska DA A prospective study of multiple protein biomarkers to predict progression in diabetic chronic kidney disease. Nephrol Dial Transplant 2014;29:2293–302. 25085239 10.1093/ndt/gfu255

[16] Liu KD Yang W Anderson AH Urine neutrophil gelatinase-associated lipocalin levels do not improve risk prediction of progressive chronic kidney disease. Kidney Int 2013;83:909–14. 23344473 10.1038/ki.2012.458 PMC3642209

[17] Zhou J Chen Y Liu Y Urinary uromodulin excretion predicts progression of chronic kidney disease resulting from IgA nephropathy. PLoS One 2013;8:e71023. 23990922 10.1371/journal.pone.0071023 PMC3750049

[18] Garimella PS Biggs ML Katz R Urinary uromodulin, kidney function, and cardiovascular disease in elderly adults. Kidney Int 2015;88:1126–34. 26154925 10.1038/ki.2015.192 PMC4653069

[19] Kidney Disease: Improving Global Outcomes (KDIGO) CKD Work Group. KDIGO 2012 clinical practice guideline for the evaluation and management of chronic kidney disease. Kidney Int Suppl 2013;3:1–50.

[20] Inker LA Schmid CH Tighiouart H Estimating glomerular filtration rate from serum creatinine and cystatin C. N Engl J Med 2012;367:20–9. 22762315 10.1056/NEJMoa1114248 PMC4398023

[21] Maple-Brown LJ Hughes JT Ritte R Progression of kidney disease in indigenous Australians: the eGFR follow-up study. Clin J Am Soc Nephrol 2016;11:993–1004. 27076636 10.2215/CJN.09770915 PMC4891751

[22] Sood MM Akbari A Manuel DG Longitudinal blood pressure in late-stage chronic kidney disease and the risk of end-stage kidney disease or mortality (Best Blood Pressure in Chronic Kidney Disease Study). Hypertension 2017;70:1210–8. 29061722 10.1161/HYPERTENSIONAHA.117.09855

[23] Mc Causland FR Claggett B Burdmann EA C-reactive protein and risk of ESRD: results from the Trial to Reduce Cardiovascular Events With Aranesp Therapy (TREAT). Am J Kidney Dis 2016;68:873–81. 27646425 10.1053/j.ajkd.2016.07.022 PMC5123931

[24] Inaguma D Imai E Takeuchi A Risk factors for CKD progression in Japanese patients: findings from the Chronic Kidney Disease Japan Cohort (CKD-JAC) study. Clin Exp Nephrol 2017;21:446–56. 27412450 10.1007/s10157-016-1309-1 PMC5486452

[25] Coresh J Turin TC Matsushita K Decline in estimated glomerular filtration rate and subsequent risk of end-stage renal disease and mortality. JAMA 2014;311:2518–31. 24892770 10.1001/jama.2014.6634 PMC4172342

[26] Chang WX Asakawa S Toyoki D Predictors and the subsequent risk of end-stage renal disease: usefulness of 30% decline in estimated GFR over 2 years. PLoS One 2015;10:e0132927. 26177463 10.1371/journal.pone.0132927 PMC4503403

[27] Mao S Zhang A Huang S The signaling pathway of uromodulin and its role in kidney diseases. J Recep Signal Trans Res 2014;34:440–4. 10.3109/10799893.2014.920029 24849497

[28] Garimella PS Katz R Ix JH Association of urinary uromodulin with kidney function decline and mortality: the health ABC study. Clin Nephrol 2017;87:278–86. 28332475 10.5414/CN109005 PMC6102560

[29] Pivin E Ponte B de Seigneux S Uromodulin and nephron mass. Clin J Am Soc Nephrol 2018;13:1556–7. 30054352 10.2215/CJN.03600318 PMC6218822

[30] Steubl D Block M Herbst V Plasma uromodulin correlates with kidney function and identifies early stages in chronic kidney disease patients. Medicine (Baltimore) 2016;95:e3011. 26962815 10.1097/MD.0000000000003011 PMC4998896

[31] Smith ER Lee D Cai MM Urinary neutrophil gelatinase-associated lipocalin may aid prediction of renal decline in patients with non-proteinuric Stages 3 and 4 chronic kidney disease (CKD). Nephrol Dial Transplant 2013;28:1569–79. 23328709 10.1093/ndt/gfs586

[32] Kim SS Song SH Kim IJ Urinary cystatin C and tubular proteinuria predict progression of diabetic nephropathy. Diabetes Care 2013;36:656–61. 23093662 10.2337/dc12-0849 PMC3579333

[33] Bhavsar NA Kottgen A Coresh J Neutrophil gelatinase-associated lipocalin (NGAL) and kidney injury molecule 1 (KIM-1) as predictors of incident CKD stage 3: the Atherosclerosis Risk in Communities (ARIC) Study. Am J Kidney Dis 2012;60:233–40. 22542304 10.1053/j.ajkd.2012.02.336 PMC3399971

[34] Peralta CA Katz R Bonventre JV Associations of urinary levels of kidney injury molecule 1 (KIM-1) and neutrophil gelatinase-associated lipocalin (NGAL) with kidney function decline in the Multi-Ethnic Study of Atherosclerosis (MESA). Am J Kidney Dis 2012;60:904–11. 22749388 10.1053/j.ajkd.2012.05.014 PMC3690926

[35] Nielsen SE Andersen S Zdunek D Tubular markers do not predict the decline in glomerular filtration rate in type 1 diabetic patients with overt nephropathy. Kidney Int 2011;79:1113–8. 21270761 10.1038/ki.2010.554

[36] Waanders F Vaidya VS van Goor H Effect of renin-angiotensin-aldosterone system inhibition, dietary sodium restriction, and/or diuretics on urinary kidney injury molecule 1 excretion in nondiabetic proteinuric kidney disease: a post hoc analysis of a randomized controlled trial. Am J Kidney Dis 2009;53:16–25. 18823687 10.1053/j.ajkd.2008.07.021 PMC3298772

[37] Tekce BK Uyeturk U Tekce H Does the kidney injury molecule-1 predict cisplatin-induced kidney injury in early stage? Ann Clin Biochem 2015;52:88–94. 24670880 10.1177/0004563214528312

[38] Ho J Tangri N Komenda P Urinary, plasma, and serum biomarkers’ utility for predicting acute kidney injury associated with cardiac surgery in adults: a meta-analysis. Am J Kidney Dis 2015;66:993–1005. 26253993 10.1053/j.ajkd.2015.06.018

[39] Schanstra JP Zurbig P Alkhalaf A Diagnosis and prediction of CKD progression by assessment of urinary peptides. J Am Soc Nephrol 2015;26:1999–2010. 25589610 10.1681/ASN.2014050423 PMC4520165

[40] Wilson FP Xie D Anderson AH Urinary creatinine excretion, bioelectrical impedance analysis, and clinical outcomes in patients with CKD: the CRIC study. Clin J Am Soc Nephrol 2014;9:2095–103. 25381342 10.2215/CJN.03790414 PMC4255402

[41] Di Micco L Quinn RR Ronksley PE Urine creatinine excretion and clinical outcomes in CKD. Clin J Am Soc Nephrol 2013;8:1877–83. 24158796 10.2215/CJN.01350213 PMC3817899

[42] Astor BC Matsushita K Gansevoort RT Lower estimated glomerular filtration rate and higher albuminuria are associated with mortality and end-stage renal disease. A collaborative meta-analysis of kidney disease population cohorts. Kidney Int 2011;79:1331–40. 21289598 10.1038/ki.2010.550 PMC3917543

