# Supplementary material for: Urinary uromodulin independently predicts end-stage renal disease and rapid kidney function decline in a cohort of chronic kidney disease patients
Source: Medicine (Baltimore). 2019 May 24;98(21):e15808. doi: 10.1097/MD.0000000000015808 (PMC6571211; doi:10.1097/MD.0000000000015808)
Supplement: Supplemental Digital Content [file medi-98-e15808-s001.doc]

Suppl. Table 1: Baseline characteristics of patients lost to follow-up (n=75)

| Variable | Values |
| --- | --- |
| Urinary UMOD (µg/ml) | 7.0 (0;37.0) |
| Age (years) | 61 (20;86) |
| Gender (male/female) | 42/33 |
| Diabetes mellitus (n (%)) | 23 (30.7) |
| CHD (n (%)) | 10 (13.3) |
| PAD (n (%)) | 2 (2.7) |
| BMI (kg/m^2^) | 25.7 (16.9;41.2) |
| Systolic BP (mmHg) | 136 (90;200) |
| Diastolic BP (mmHg) | 80 (55;130) |
| eGFR (ml/min/1.73 m^2^) | 44.8 (4.1;145.3) |
| Proteinuria (mg/g Cr) | 290 (0;8721) |
| C-reactive protein (mg/dl) | 0.3 (0;14) |
| ACEI/ARB (n (%)) | 46 (61.3) |
| Aldosterone antagonist (n (%)) | 2 (2.7) |
| Bicarbonate (n (%)) | 10 (13.3) |
| Erythropoetic agent (n (%)) | 1 (1.3) |
| Active vitamin D (n (%)) | 18 (24.0) |
| UA lowering agent (n (%)) | 11 (14.7) |
| Phosphate binders (n (%)) | 14 (18.7) |

Values in median (minimum;maximum) for continuous variables; UMOD = uromodulin; eGFR = estimated glomerular filtration rate (CKD-EPI_creatinine/cystatin C_); CHD = coronary heart disease; PAD = peripheral arterial disease; BMI = body-mass-index; BP = blood pressure; ACEI = angiotensin-converting-enzyme-inhibitor; ARB = angiotensin-receptor-blocker; UA = uric acid;

Suppl. Table 2: Evaluation for interactions between uUMOD quartiles and eGFR/proteinuria in multivariable cox regression analysis with the composite endpoint ESRD/25%-eGFR decline

| Interaction term | HR (95%-CI) | p-value |
| --- | --- | --- |
| Q4:eGFR | Reference |  |
| Q3:eGFR | 1.003 (0.996-1.009) | 0.386 |
| Q2:eGFR | 1.003 (0.997-1.010) | 0.299 |
| Q1:eGFR | 1.003 (0.997-1.010) | 0.346 |
| Q4:proteinuria | Reference |  |
| Q3:proteinuria | 1.040 (0.989-1.086) | 0.138 |
| Q2:proteinuria | 0.969 (0.927-1.013) | 0.160 |
| Q1:proteinuria | 0.937 (0.873-1.006) | 0.073 |

uUMOD=urinary uromodulin; eGFR=estimated glomerular filtration rate in ml/min/1.73 m^2^; ESRD=end-stage renal disease; HR=hazard ratio; CI=confidence interval; Q1=1. uUMOD quartile (≤ 2.6 µg/ml); Q2=2. uUMOD quartile (2.6-4.75 µg/ml); Q3=3. uUMOD quartile (4.75-11.45 µg/ml); Q4=4. uUMOD quartile (≥ 11.45 µg/ml); proteinuria in mg/g creatinine; multivariable model adjusted for systolic blood pressure, C-reactive protein, oral active vitamin D and phosphate binder use and eGFR/proteinuria vice versa.
